# Neural Correlates of the In-Group Memory Advantage on the Encoding and Recognition of Faces

**DOI:** 10.1371/journal.pone.0082797

**Published:** 2013-12-17

**Authors:** Grit Herzmann, Tim Curran

**Affiliations:** 1 Department of Psychology, The College of Wooster, Wooster, Ohio, United States of America; 2 Department of Psychology and Neuroscience, University of Colorado Boulder, Boulder, Colorado, United States of America; University of Leuven, Belgium

## Abstract

People have a memory advantage for faces that belong to the same group, for example, that attend the same university or have the same personality type. Faces from such in-group members are assumed to receive more attention during memory encoding and are therefore recognized more accurately. Here we use event-related potentials related to memory encoding and retrieval to investigate the neural correlates of the in-group memory advantage. Using the minimal group procedure, subjects were classified based on a bogus personality test as belonging to one of two personality types. While the electroencephalogram was recorded, subjects studied and recognized faces supposedly belonging to the subject’s own and the other personality type. Subjects recognized in-group faces more accurately than out-group faces but the effect size was small. Using the individual behavioral in-group memory advantage in multivariate analyses of covariance, we determined neural correlates of the in-group advantage. During memory encoding (300 to 1000 ms after stimulus onset), subjects with a high in-group memory advantage elicited more positive amplitudes for subsequently remembered in-group than out-group faces, showing that in-group faces received more attention and elicited more neural activity during initial encoding. Early during memory retrieval (300 to 500 ms), frontal brain areas were more activated for remembered in-group faces indicating an early detection of group membership. Surprisingly, the parietal old/new effect (600 to 900 ms) thought to indicate recollection processes differed between in-group and out-group faces independent from the behavioral in-group memory advantage. This finding suggests that group membership affects memory retrieval independent of memory performance. Comparisons with a previous study on the other-race effect, another memory phenomenon influenced by social classification of faces, suggested that the in-group memory advantage is dominated by top-down processing whereas the other-race effect is also influenced by extensive perceptual experience.

## Introduction

When a friend introduces us to a stranger, there are multiple factors that influence how well we will remember the newly learned face. The situation of the encounter as well as our own abilities to remember any face will affect subsequent memory. Research has shown that the match between certain characteristics of the encountered person and us will determine the fate of the memory too. The best known example is perhaps the other-race effect, the phenomenon that people recognize faces from their own-race much better than faces from a different race [1], [2]. There are other physiognomic features that can bias face recognition like age or sex. In addition to these clearly visible characteristics, recent research has shown that belonging to the same group as an encountered person affects face memory in a similar way: faces from people of the same group (or in-group) are recognized more accurately than faces from people of another group (or out-group) [3]–[7]. Group membership can, for example, mean being affiliated with the same university or sports team but it can also include experimentally created minimal groups which are based on bogus personality [3], [8], [9] or bogus cognitive tests [10] or simply on telling subjects that they now belong to a specific group [5], [6], [11]. Here, we investigate the neural processes that underlie the in-group memory advantage that arises from mere group membership. For this purpose, we used the minimal group procedure [12].

In the minimal group procedure, subjects are arbitrarily assigned to one of two distinct groups that sometimes only exist in the experiment context. Nevertheless, subjects believe that these groups are real. In the present study, for example, subjects completed a bogus personality test which grouped them in either the yellow or blue personality type. After this, subjects completed a face recognition experiment in which they studied and recognized faces from the yellow and blue personality type. As done in previous research, the assignment of the subjects and the stimuli to the personality types was completely arbitrary. Nevertheless, the mere group membership influenced neural processes of memory encoding and memory retrieval as the present study showed.

Previous research using the minimal group procedure has repeatedly shown that in-group faces are recognized more accurately than out-group faces [3]–[8]. These studies proposed that in-group and out-group faces are differently processed during memory encoding but not recognition [8]. To explain the in-group memory advantage, they draw on the in-group/out-group model by Sporer [13] and suggest that in-group faces are processed, by default, more holistically which leads to superior recognition whereas social categorization disrupts default holistic processing and leads to poor out-group recognition [3]. Indeed, Hugenberg and Corneille [14] used the composite face effect to show that faces of in-group members (i.e., members of the same university) were processed more holistically than faces of out-group members (from a different university). In another study, Young and Hugenberg [9] showed that emotion recognition of in-group faces (from the same bogus personality type as the subjects) but not out-group faces was impaired when turning the stimulus upside-down thus using the so-called inversion effect to show that in-group faces were processed more holistically. Alternative to Sporer’s theory, Levin’s theory [15], [16] is used to explain the in-group memory advantage of the minimal group procedure by assuming that subjects differently attend to in-group and out-group members, thereby focusing on individuating features of the faces of in-group members and concentrating on category-specific information or broad similarity among out-group members [3], [4]. Individuation is assumed to lead to better recognition than concentrating on category-specific information. In support of this theory, Van Bavel and Cunningham [5] found that in-group faces attracted more attention during memory encoding and that attention mediated subsequent recognition of in-group and out-group members. In their experiment, subjects were allowed to flip through the to-be-learned faces in a self-paced manner. The time spent encoding each individual faces was taken as a measure of how much attention was allocated to in-group and out-group members. Subjects spent more time looking at in-group than out-group faces and remembered in-group faces more accurately. The same study also showed that subjects who identified more with their respective group showed a larger in-group memory advantage. The authors suggested that perceptual processing together with motivational factors like identification with and value of the group membership shaped face recognition of in-group faces [5]. Taken together, these studies provided evidence that in-group faces received more attention and more holistic processing during memory encoding. In addition, individual differences in the in-group memory advantage were found because motivational factors and the feeling of group membership showed a positive correlation to the size of the in-group memory advantage.

Neuroscientific methods like fMRI and event-related potentials (ERP) have been used to start to understand the neural processes influencing how faces of in-group members are perceived. Even though none of these studies investigated memory processes, they still contribute valuable information to the background of the present study. Two fMRI studies used categorization tasks in a blocked design to measure brain activity for in-group and out-group faces. These studies found that the amygdala, fusiform gyri, fusiform face area, orbitofrontal cortex, and dorsal striatum were more activated for in-group than out-group faces [6], [11] suggesting that these brain areas are susceptible to top-down processing of group membership. Interestingly, some of these areas like the fusiform gyrus, the amygdala, and orbitofrontal gyrus play a role in face recognition and might thus contribute to the in-group memory advantage. The fusiform face area, for example, may play a role in encoding in-group members more individually [6]. The amygdala and orbitofrontal cortex may be involved in segregating more relevant and salient in-group from less relevant out-group faces [11]. These fMRI studies did also find individual differences in the processing of in-group faces. Activity in the orbitofrontal cortex and fusiform face area were both positively correlated with a processing advantage for in-group faces [6], [11].

A recent ERP study concentrated on perceptual ERP-components to show that group membership influenced very early visual processing. Ratner and Amodio [10] investigated the structural encoding of faces by comparing the N170 to in-group and out-group faces, indicated by background color. In-group faces elicited a larger N170 than out-group faces which suggested that in-group faces were more readily processed and received more processing resources during early visual encoding. The authors concluded that this result pointed to an early top-down influence of group membership [10].

In summary, previous studies using the minimal group procedure have shown a clear memory advantage for in-group over out-group faces. Facilitations in perceptual processing and memory encoding are assumed to account for this advantage. FMRI studies showed that brain areas in the face recognition network were influenced by mere group membership, and ERPs yielded evidence for an early influence of group status on perceptual processing. No study has so far investigated the memory processes of the in-group memory advantage. The present study therefore aimed to identify the neural processes of memory encoding and memory retrieval that correlate with enhanced recognition memory performance for in-group as compared to out-group faces. Specifically, the present study focused on the two sub-processes of recognition memory: familiarity (i.e., a face feels familiar) and recollection (i.e., a face is remembered with details from the study episode) [17].

To investigate the neural correlates of the in-group memory advantage, we used ERPs related to memory encoding and memory retrieval. The ERP difference due to memory (Dm) is taken to reflect memory encoding. It is measured in the study phase of a memory task [18] and obtained by sorting study-phase ERPs according to the participant’s memory performance in the subsequent recognition test. Prefrontal, medial-temporal, and parietal areas have been identified as brain regions related to subsequent memory effects in fMRI studies [19]. The FN400 and parietal old/new effect are ERP correlates of memory retrieval. The FN400 distinguishes hits from correct rejections without being influenced by the recollection of details from the study episode [20]. It is thus taken as a correlate of familiarity processes and likely generated in the prefrontal cortex [21]. The parietal old/new effect varies with the amount of recollected information from the study episode [22], [23] and thus measures recollection processes. It is most likely generated in the parietal cortex [21]. Previous fMRI studies showed that the fusiform face area, amygdala, and orbitofrontal cortex were more activated for in-group than out-group faces [6], [11]. Together with the finding of allocating more attention to in-group faces during memory encoding [5], we expected that we would find larger Dms for in-group than out-group faces during the study phase showing that in-group faces receive more attention and memory encoding activity. Faces that are encoded with more attention and are recognized more accurately can be assumed to be associated with richer memory representations. We therefore expected the parietal old/new effect to be larger for in-group than out-group faces. If group membership would influence familiarity processes, we would also expect to see an effect of group membership on the FN400.

The present study showed a small in-group memory advantage in performance measures across all subjects and a correlate of this effect during memory retrieval. The retrieval of in-group faces was associated with a larger parietal old/new effect whereas the old/new effect for out-group faces was smaller and extended to frontal brain areas. No other correlates of the minimal group procedure were found across all subjects. In accord with previous research that showed individual differences in the in-group memory advantage [5], [6], [11], we too observed large individual differences. To further investigate the neural correlates of the minimal group effect, we used the behavioral in-group memory advantage (based on measures of P(*A*) using the receiver operating characteristic curve) as covariate in multivariate analyses of covariance (MANCOVAs) of the ERP data. These analyses showed that for subjects exhibiting a larger in-group memory advantage in-group faces received more neural resources during memory encoding (300 to 1000 ms) and elicited frontal brain activation early during the retrieval process (300 to 500 ms). The present study also analyzed perceptual ERPs (P100, N170, P200, and N250). No significant interactions with group membership were found. Consequently, these analyses and results are not reported here.

## Methods

### 2.1 Subjects

Forty-five (34 women; mean age: 19.8 ± 2.6, range: 18–29 years) healthy, young, right-handed, adults volunteered in this study. The majority of subjects were Caucasian (40), two were Asian, and three were Hispanic. This study investigated face memory and used Caucasian faces. Given the other-race effect in face recognition, it might seem surprising that Non-Caucasian subjects were included in the study. However, here we are interested in the effects of social categorization, which was manipulated within subjects. Each subject was therefore his or her own control subject, and the results are independent from any possible influence of an other-race effect.

All subjects had normal or corrected to normal vision. The study was approved by the University of Colorado Boulder Institutional Review Board (IRB number 0309.26) and was conducted in accordance with the Declaration of Helsinki. All participants gave written informed consent and were paid for their participation.

### 2.2 Stimuli and apparatus

Stimuli were digital, color portrait photographs of 640 Caucasian faces (Color FERET database) [24]. Faces showed neutral or smiling expression. Half of the faces were female. Stimuli were cropped (5.3 cm×7.0 cm) to show only the face and hair but no necks or clothing. All face stimuli were duplicated. Then, one set was placed on a yellow background and the other on a blue background. Stimuli were shown at a viewing distance of one meter on a 17-inch flat-panel LCD monitor. Stimulus presentation (and EEG recording) was time-locked to the refresh point of the monitor.

### 2.3 Procedure

The study consisted of one three-hour session. The session started with a bogus personality test that associated subjects with either the blue or yellow personality type as done in previous studies (e.g., [3], [9]). Then subjects studied 320 faces, half from the blue and the other half from the yellow personality type as indicated by the background color. The study phase was repeated to ensure high enough memory performance in the subsequent recognition test. During the test phase, all 320 studied faces were presented together with 320 completely new distracter faces. Again, half of the faces were from the blue and half from the yellow personality type as indicated by the background color. Assignment of a given face stimulus to personality type and target/distracter status was completely randomized. The EEG was recorded during study and test. At the end of the session, subjects completed a questionnaire about the affiliation they felt toward their personality type and were debriefed.

At the beginning of the experimental session, subjects received the following instructions, closely following previous studies [3], [8], [9]:

*In this experiment, we want to investigate how personality influences memory for faces. You will first complete a personality test. It associates personality types with colors. The test will determine if you have a blue or a yellow personality type. You will then complete a memory test that uses faces of people with either blue or yellow personality types.*

Following these instructions, subjects completed the bogus personality test on the computer. The personality test (see File S1) had 44 questions, partly drawn from the Big Five questionnaire [25]. Each question was individually presented on the screen. Subjects responded using a 5-point rating scale (1-disagree strongly to 5-agree strongly). After the last response, the computer supposedly analyzed the answers while presenting a countdown from 10 to 1 and determined the personality type. In reality, none of the responses to the personality test were recorded. The personality type was predetermined and assigned counterbalanced across subjects.

After the personality type of the subject was determined, they were given a sign that stated their personality type. In addition to the personality type, subjects received the following feedback as done in previous studies [3], [8], [9].

*This personality measure has been found to be very good at predicting future success both socially and monetarily. The measure itself is often used by businesses and organizations as a means of identifying strong candidates for competitive positions. Further, psychologists who study relationships often use this personality inventory to identify future success in relationships. As a member of the [blue or yellow] personality type, you share many similar qualities with other members of your group. After completing this experiment, you will learn more details about the [blue or yellow] personality type.*

Subjects were not given any further information on the personality type. After receiving the sign that stated the personality group, subjects were instructed for the memory experiment. The study phase consisted of two runs, each showing the same face stimuli but in a different, randomized order. In each run, subjects studied 160 faces each from the blue and yellow personality type intermixed in 16 blocks. Short breaks were allowed after every 20 faces. In the study phase, each face was presented for two seconds. Subjects were told to memorize all stimuli as well as possible and to make attractiveness ratings (1 =  very unattractive to 7 =  very attractive) without time limit on a computer keyboard to foster memory encoding. The prompt for the attractiveness rating appeared immediately after the stimulus had disappeared to separate memory encoding from attractiveness ratings and keep the presentation time for all items constant. One second after the response, the next stimulus was presented. A fixation cross was shown in the response-to-stimulus interval. The whole study phase lasted between 70 to 80 minutes.

The recognition test started about 5 to 10 minutes after the end of the study. All 320 studied items intermixed with 160 new faces from the blue and 160 new faces from the yellow personality type were tested. Short breaks were allowed after every 20 faces. Each stimulus was presented for 1.5 seconds. The response options then appeared below the stimulus, and subjects were asked, without time limit, to make memory judgments by pressing the corresponding key on a computer keyboard. They were told to judge the face as “recollected” when they could remember the presented face together with specific details about learning this face in the study phase (such as a thought that came to mind or something that happened in the room). In the case that they did not recollect a face, they were asked to rate its familiarity. They were told to use “definitely familiar” or “maybe familiar” if they believed that they had seen the face in the study phase but could not consciously remember anything particular about its appearance or the experience of learning it. “Maybe unfamiliar” or “definitely unfamiliar” were to be used if they did not recognize the face from the study phase [26]. Before the beginning of the study phase, participants practiced making recollect/familiar judgments to verify, as judged by the experimenter, that they fully understood the differences between the meanings of these memory judgments.

After the recognition test, subjects completed a questionnaire about the affiliation they felt toward their personality type (File S2). Subjects were then debriefed about the bogus personality test and dismissed.

### 2.4 Performance measurement

For recognition memory performance, we considered percent of hits, percent of false alarms, the area below the receiver operating characteristic (ROC curve, P(*A*), [27]), and hits minus false alarms of “recollect” and “familiar” responses. ROC curves were computed from all five possible response bins, with “recollect” responses treated as reflecting higher confidence than “definitely familiar” responses. We interpret raw “recollect” judgments as corresponding to recollection. The raw “familiar” condition (i.e., “maybe familiar” and “definitely familiar”) cannot be taken as a direct reflection of dual-process familiarity because these responses are contingent upon non-recollection. Using the so-called independent remember-know procedure (IRK, [17]), familiarity hits and false alarms were calculated as the probability of responding “familiar” to an item provided that the item was not given a “recollect” response (i.e., for hit rates and false alarms, respectively, IRK “familiar”  =  “familiar”/(1-“recollect”)).

### 2.5 Event-related potential recording and measurement

**Figure 1:**
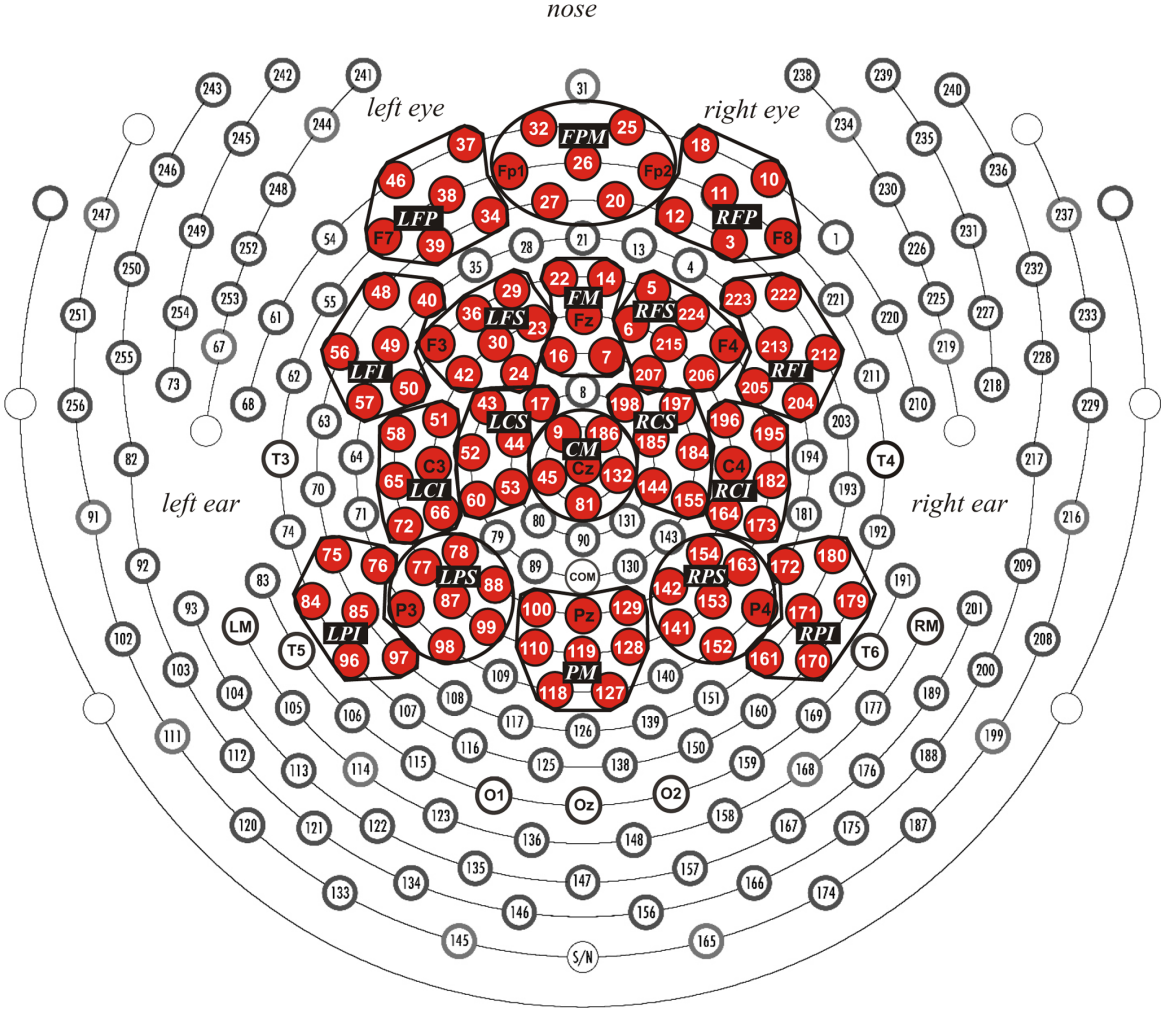
Geodesic sensor net layout. Electrode sites are numbered. Red clusters are regions of interest included in analyses. LFP  =  left frontal-polar, FPM  =  frontal-polar medial, RFP  =  right frontal-polar, LFI  =  left frontal inferior, LFS  =  left frontal superior, FM  =  frontal medial, RFS  =  right frontal superior, RFI  =  right frontal inferior, LCI  =  left central inferior, LCS  =  left central superior, CM  =  central medial, RCS  =  right central superior, RCI  =  right central inferior, LPI  =  left parietal inferior, LPS  =  left parietal superior, PM  =  parietal medial, RPS  =  right parietal superior, RPI  =  right parietal inferior.

The EEG was recorded in the study and recognition test phase with a 256-channel Geodesic Sensor Net™ (HGSN 256 v. 1.0, [28], Fig. 1) connected to an AC-coupled, high-input impedance amplifier (200 MΩ, Net Amps™, Electrical Geodesics Inc., Eugene, OR). Amplified analog voltages (0.1–100 Hz bandpass) were digitized at 250 Hz. The recording reference was the vertex channel (Cz). Individual sensors were adjusted until impedances were less than 50 kΩ.

Epochs of 1100 ms for study-phase items and 1300 ms for test-phase items, each starting 100 ms before stimulus onset, were generated offline from the continuous record. Horizontal and vertical eye movements were corrected using the ocular correction ICA transformation in Brain Vision Analyzer 2.0.1 (Brain Products GmbH, Munich, Germany). Trials with non-ocular artifacts were discarded. ERPs were aligned to a 100-ms baseline before target onset, averaged separately for each channel and condition, digitally low-pass filtered at 40 Hz, and recalculated to average reference. A minimum of 15 trials per condition was ensured for each subject.

**Figure 2:**
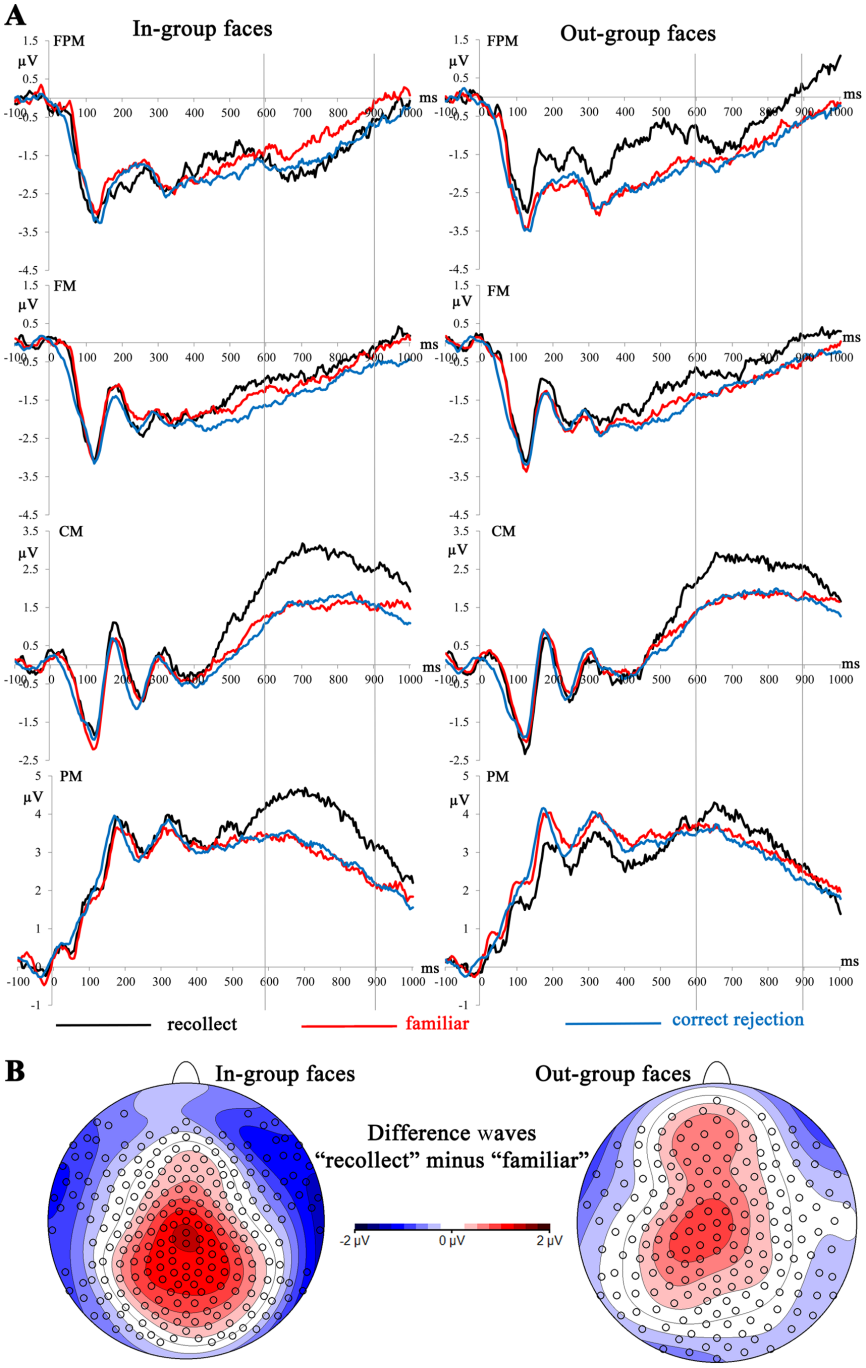
A) Mean amplitudes from the test phase depicting recognition-related brain activation for “recollected” and “familiar” old faces and correctly rejected new faces for in-group and out-group faces. Vertical lines highlight the time segment from 600 to 900 ms used for statistical analysis of the parietal old/new effect. See Figure 1 for abbreviations of regions of interest and their locations. B) Voltage maps of ERP difference waves between “recollected” and “familiar” conditions showing the parietal old/new effect at 600-900 ms for in-group and out-group faces. Spherical spline interpolation was used.

**Figure 3:**
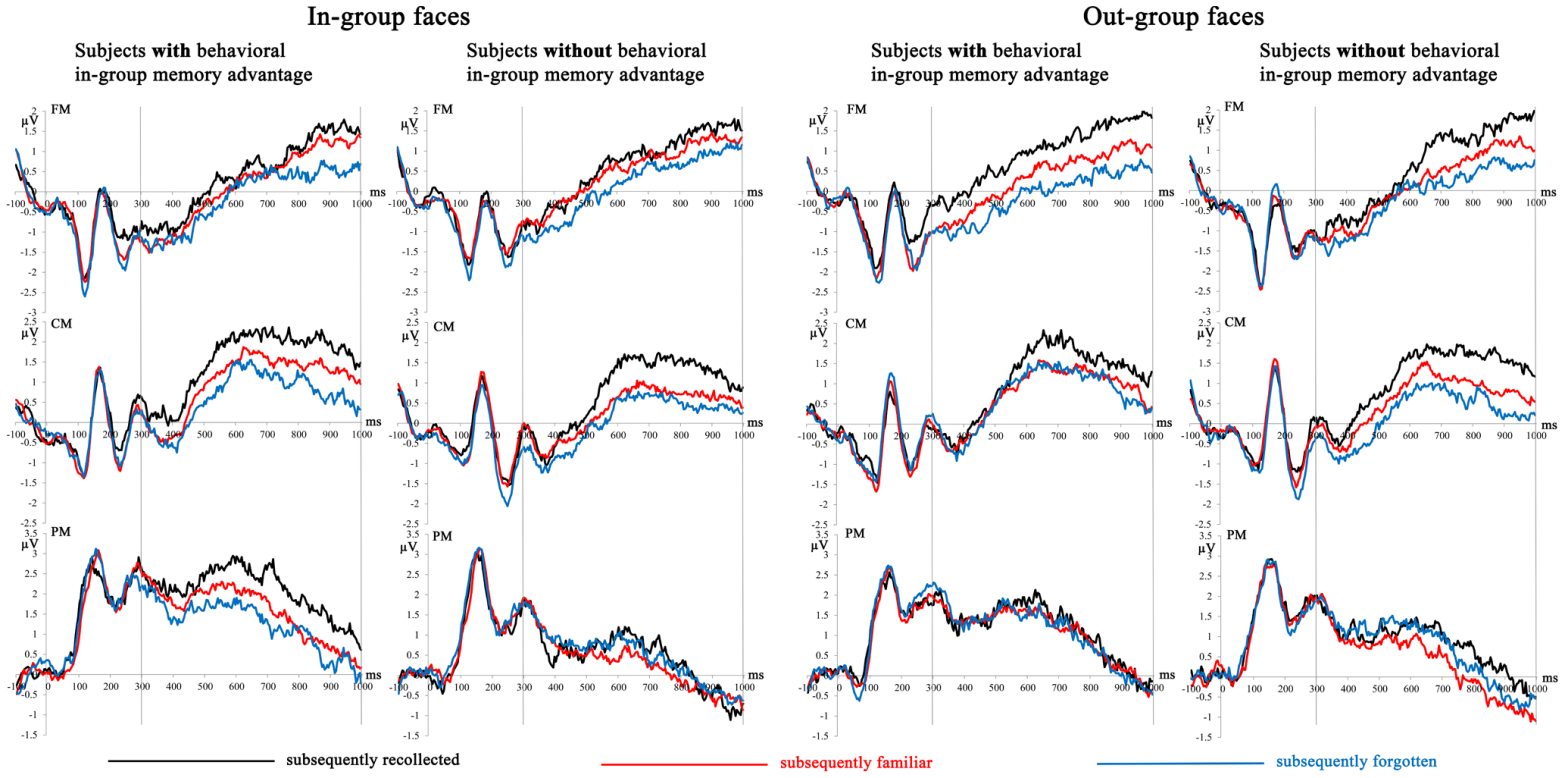
Mean amplitudes from the first run of the study phase depicting encoding-related brain activation (Dms) for subsequently “recollected,” “familiar,” and forgotten in-group and out-group faces for subjects with and without a behavioral in-group memory advantage. See Figure 1 for abbreviations of regions of interest and their locations.

**Figure 4:**
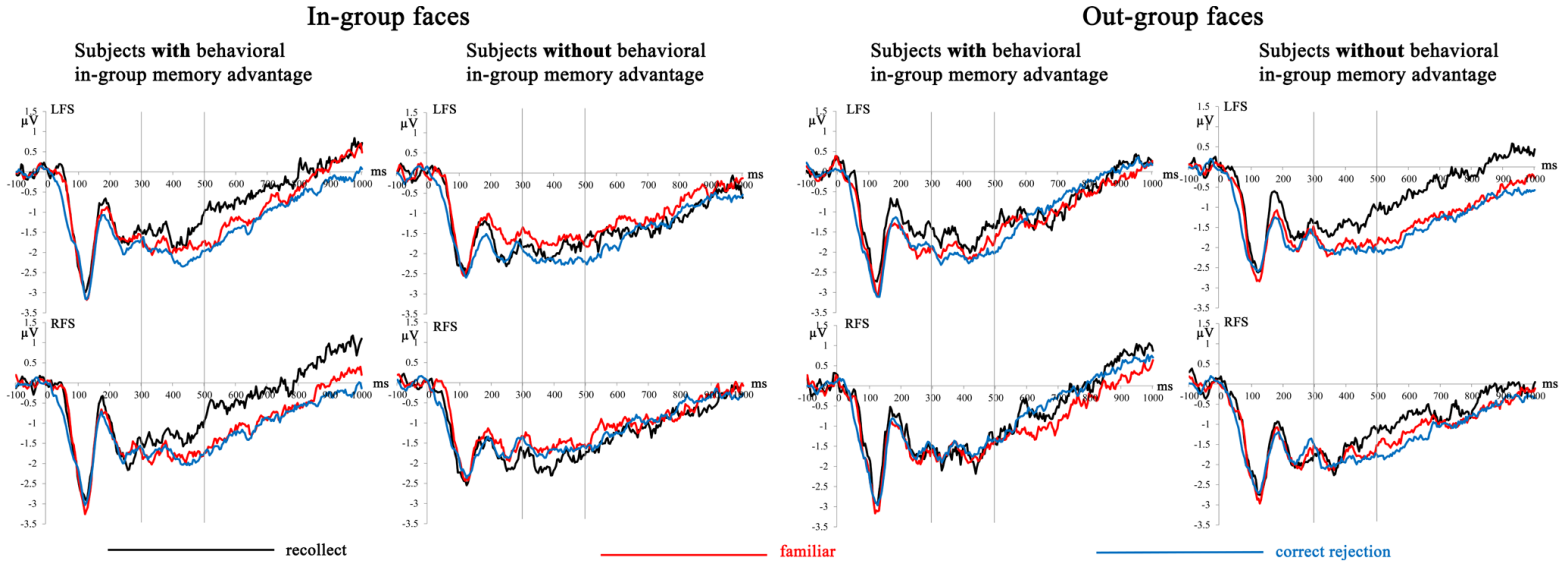
Mean amplitudes from the test phase depicting recognition-related brain activation for “recollected” and “familiar” old faces and correctly rejected new in-group and out-group faces for subjects with and without a behavioral in-group memory advantage. Vertical lines highlight the time segment from 300 to 500 ms used for statistical analysis of the FN400 old/new effect. See Figure 1 for abbreviations of regions of interest and their locations.

**Figure 5:**
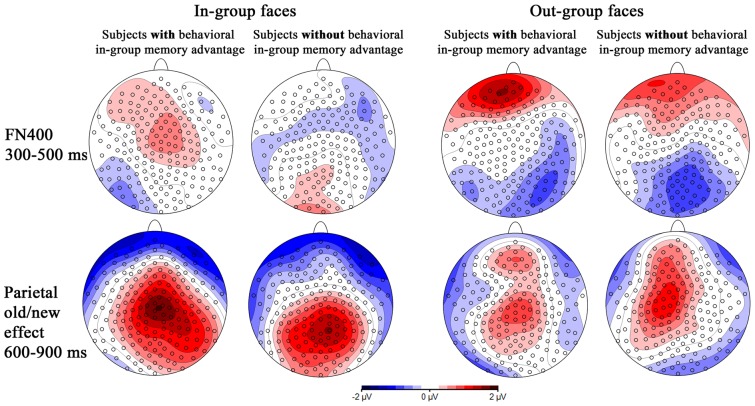
Voltage maps of ERP difference waves between “recollected” and “familiar” conditions comparing the FN400 at 300-500 ms and the parietal old/new effect at 600-900 ms for in-group and out-group faces in subjects with and without a behavioral in-group memory advantage. Spherical spline interpolation was used.

Time segments and regions of interest (ROIs) were defined according to visual inspection (Fig. 2–5) and previous research [29], [30] (Fig. 1). Mean amplitudes were computed by averaging the channels within each ROI for each condition and subject. The Dm was measured between 300 and 1000 ms. The FN400 was measured between 300 and 500 ms and the parietal old/new effect between 600 and 900 ms. ROIs for the Dm were five channel groups each over frontal, central, and parietal regions (frontal: LFI, LFS, FM, RFS, RFI; central: LCI, LCS, CM, RCS, RCI; parietal: LPI, LPS, PM, RPS, RPI; Fig. 1). ROIs for the FN400 were the left and right frontal superior channel groups (LFS, and RFS, Fig. 1). ROIs for the parietal old/new effects were the medial as well as the left and right superior channel groups over fronto-polar, frontal, central, and parietal regions (FPM, FPL, FPR; FM, LFS, RFS; CM, LCS, RCS; PM, LPS, and RPS, Fig. 1).

### 2.6 Data analysis

Behavioral and ERP data was analyzed in two ways. First, as generally done, the in-group memory advantage was investigated across all subjects. Second, a continuous measure of the behavioral in-group memory advantage was used in regression analyses to further determine how group membership of the faces affected familiarity and recollection as well as neural correlates of memory encoding and retrieval. In these regression analyses, the behavioral in-group memory advantage was measured as the difference in memory performance (quantified as P(*A*)) between in-group and out-group faces.

For behavioral data, the highest-level analyses were t-tests between the levels of the within-subject factor stimulus group (in-group, out-group).

For ERP measures, ANOVAs were calculated for two different contrasts of the factor memory judgment. The contrast that tested recollection processes was calculated between ERPs to “recollected” vs. “familiar” faces. The contrast that tested familiarity processes was calculated between ERPs to “familiar” and forgotten faces, in the case of the Dm, or correct rejections, in the case of old/new effects. Only hits were included as “recollected” and “familiar” items. In addition to the factor memory judgment, the analyses of Dm, FN400, and parietal old/new effects included other within-subject factors. The analyses of the Dm included the within-subject factors run (first and second run of the study phase), stimulus group (in-group, out-group), frontal-parietal (anterior to posterior gradient of ROIs: frontal, central, parietal, see 2.5 and Fig. 1), and left-right (laterality gradient of ROIs: inferior left, superior left, medial, superior right, inferior right, see 2.5 and Fig. 1). Analyses of the FN400 included the within-subject factors stimulus group (in-group, out-group) and ROI (left and right frontal superior ROI). Analyses of the parietal old/new effect included the within-subject factors stimulus group (in-group, out-group), frontal-parietal (anterior to posterior gradient of ROIs: fronto-polar, frontal, central, parietal, see 2.5 and Fig. 1), and left-right (laterality gradient of ROIs: superior left, medial, superior right, see 2.5 and Fig. 1).

In addition to these general analyses, additional analyses were calculated to further investigate the in-group memory advantage. These MANCOVAs used the same within-subject factors as described above and added the behavioral in-group memory advantage as covariate.

Post-tests that followed up on any significant main effect or interaction were Bonferroni-corrected for multiple comparisons. All p-values associated with more than one degree of freedom were corrected according to the [31] procedure for sphericity violations but we report uncorrected degrees of freedom.

## Results and Discussion

### 3.1 General analysis across all subjects

**3.1.1 Behavioral data.** Table 1 summarizes behavioral indicators of memory performance and effect sizes (as Cohen’s d) for the in-group memory advantage (i.e., the difference between in-group and out-group faces) across all subjects. The only significant in-group memory advantage was observed for the area below the ROC curve (P(*A*)), *t*(44)  = 2.09, *p* = .043, d = .14.

**Table 1:** Performance data comparing in-group and out-group faces.

	In-group faces		Out-group faces		
	*M*	*SD*	*M*	*SD*	Cohen’s d
P(*A*)	0.80	0.08	0.78	0.08	0.14
c	0.03	0.42	0.02	0.42	0.02
Hit minus false alarm “recollect”	0.26	0.17	0.25	0.16	0.06
Hit “recollect”	0.28	0.18	0.28	0.18	0.00
False alarm “recollect”	0.02	0.05	0.03	0.04	0.02
Hit minus false alarm IRK “familiar”	0.36	0.16	0.35	0.16	0.06
Hit IRK “familiar“	0.61	0.18	0.61	0.18	0.00
False alarm IRK “familiar”	0.25	0.14	0.26	0.15	0.07

Effect size is given as Cohen’s d.

There was no difference between in-group and out-group faces in attractiveness ratings made during memory encoding, *p*s >.20; nor where there reaction time differences in these ratings, *p*s >.56.

**3.1.2 ERP data.** For the Dm in the study phase and the FN400 in the test phase, no differences between memory encoding of in-group and out-group faces were observed.

A significant difference between in-group and out-group faces was found for the parietal old/new effect contrasting “recollect” and “familiar” memory judgments during the recognition test. The frontal-parietal x stimulus group x memory judgment interaction, *F*(3,129)  = 3.55, *p* = .043, showed that the distribution of old/new effects was different for in-group and out-group faces (Fig. 2). Whereas in-group faces showed the typical parietal distribution (Panel B in Figure 2), old/new effects for out-group faces were generally smaller and more widespread across the scalp. Post-tests for each level of the factor frontal-parietal showed that old/new effects for out-group faces were more positive over frontal-polar regions (FPM in Panel A of Fig. 2), *F*(1,43)  = 6.98, *p* = .022, whereas old/new effects for in-group faces tended to be more positive over parietal regions (PM in Panel A of Fig. 2), *F*(1,43)  = 2.81, *p* = .101. In-group and out-group faces did not differ over frontal and central regions.

**3.1.3 Discussion of general analysis across all subjects.** As reported in previous studies, participants were better in recognizing in-group than out-group faces [3]–[8]. The effect size of the present minimal group effect was very small. Possibly because of this small effect size, we were unable to identify whether the minimal group effect was due to an advantage in recollection or familiarity.

The ERP results provided more clarity and showed that recollection but not familiarity was affected by group membership. The parietal old/new effect, thought to reflect recollection, was modulated by the group status of the faces. The parietal old/new effect was larger and showed a more typical, parietal distribution for in-group as compared to out-group faces. This finding together with the small in-group memory advantage found in the behavioral data suggests that in-group faces were retrieved more accurately and together with more details from the study episode. The old/new effect for out-group faces extended to fronto-polar regions, where it was more positive than the old/new effect for in-group faces. This finding suggests that additional brain regions were involved in retrieving memory information about out-group faces. The pattern of parietal old/new effects for in-group and out-group faces showed some similarity to our previous finding for own-race and other-race faces [30]. There, own-race faces, similar to in-group faces in the present study, showed a typical parietal old/new effect, whereas other-race faces, as out-group faces here, yielded an old/new effect that also spread to frontal areas. We assumed that for other-race faces it was necessary to engage additional, frontal brain regions in order to remember these faces accurately. Despite the additional neural resources, memory performance for other-race faces was less accurate than for own-race faces [30]. Race is only one aspect used when categorizing faces as in-group and out-group members. The comparable results of the previous and the present study suggest that other aspects of face classification might have very similar effects on memory retrieval as the race of a face (see also the General Discussion).

Although the present study found an in-group memory advantage in the area below the ROC curve and the parietal old/new effect, the effect sizes were very small. Indeed only 22 out of 45 subjects showed a behavioral in-group memory advantage. To further elucidate the in-group memory advantage, we used the difference between in-group and out-group faces in the area below the ROC curve as covariate in MANCOVAs that investigated behavioral measures of familiarity and recollection and neural correlates of memory encoding and retrieval.

### 3.2 Analyses with continuous measure of behavioral in-group memory advantage

Subjects differed in the size of their behavioral in-group memory advantage. Here we report MANCOVAs that used a continuous measure of the in-group memory advantage as covariate. Although all of the following analyses used the continuous measure of the in-group memory advantage, we illustrate the results (Table 2 and Figures 3-5) by dividing the subject pool into the 22 subjects with an in-group memory advantage and the 23 subjects without an in-group memory advantage.

**Table 2:** Performance data separately for subjects with and without an in-group memory advantage.

	Subjects with a positive in-group memory advantage (based on P(*A*))					Subjects with no or a negative in-group memory advantage (based on P(*A*))				
	In-group faces		Out-group faces			In-group faces		Out-group faces		
	*M*	*SD*	*M*	*SD*	Cohen’s d	*M*	*SD*	*M*	*SD*	Cohen’s d
Hit minus false alarm “recollect”	0.26	0.17	0.23	0.15	0.17	0.25	0.17	0.26	0.17	0.06
Hit “recollect”	0.29	0.10	0.26	0.18	0.21	0.28	0.17	0.29	0.18	0.06
False alarm “recollect”	0.03	0.05	0.03	0.05	0.00	0.03	0.04	0.03	0.04	0.00
Hit minus false alarm IRK “familiar”	0.36	0.17	0.30	0.15	0.37	0.37	0.16	0.40	0.16	0.19
Hit IRK “familiar“	0.61	0.16	0.57	0.15	0.26	0.62	0.20	0.65	0.18	0.16
False alarm IRK “familiar”	0.25	0.15	0.27	0.16	0.13	0.25	0.13	0.25	0.14	0.00

The in-group memory advantage was determined by a median split of P(*A*).

**3.2.1 Behavioral data.** Before considering the behavioral results of the MANCOVAs, we want to clearly state that these analyses are done with measures that depend on each other. The covariate in these analyses represents a measure of overall memory performance (i.e., area below the ROC curve, P(*A*)). The measures of familiarity and recollection are a subset from the overall measure. Because of this close relationship of the measures involved, the results should be interpreted with caution. Nevertheless, we believe that these analyses are informative and will provide valuable information about the effect of social categorization of faces on familiarity and recollection. In addition, these behavioral analyses will be helpful when interpreting the ERP results of the MANCOVA.

Table 2 presents the behavioral indicators of memory performance and the effect sizes as Cohen’s d for the in-group memory advantage (i.e., the difference between in-group and out-group faces) for subjects with and without an in-group memory advantage. Significant covariations of the behavioral in-group memory advantage were found for hit rates and hits minus false alarms of recollection and familiarity measures. The larger the in-group P(*A*) memory advantage, the more accurate were “recollect” judgments, measured as hits minus false alarms, *F*(1,43)  = 10.3, *p* = .003, and “familiar” judgments, measures as hits minus false alarms, *F*(1,43)  = 43.0, *p* <.0001, to in-group as compared to out-group faces. These effects were caused by more accurate hit rates for in-group than out-group faces, *F*s(1,43)  = 9.6 and 30.2, *p*s = .003 and.0001, for hits “recollect” and hits “familiar,” respectively. No differences were found for false alarms.

The behavioral in-group memory advantage did not interact significantly with the attractiveness ratings made during memory encoding or the felt affiliation (i.e. the average of all items in the questionnaire, File S2) with the personality type, all *p*s >.23. The in-group memory advantage did also not interact with the age, *p* >.70, race, *p* >.50, or sex of the subjects, *p* >.90.

**3.2.2 ERP data in the study phase.** MANCOVAs of the recollection-related Dm (the difference between subsequently “recollected” and subsequently “familiar” faces) yielded a significant interaction of the behavioral in-group memory advantage and the run x stimulus group x memory judgment interaction, *F*(2,84)  = 5.24, *p* = .027. The MANCOVA conducted on the first run alone showed that the behavioral in-group memory advantage interacted differently with Dms for in-group and out-group faces, *F*(2,84)  = 8.09, *p* = .014. In the first run of the study phase, we found the following correlations: the larger the behavioral in-group memory advantage, the more positive the recollection-related Dm for in-group faces (*r* = .19, *p* = .21) and the less positive the recollection-related Dm for out-group faces (*r* = –.22, *p* = .16). Figure 3 illustrates this finding. The recollection-related Dm for in-group faces is larger for subjects with than for those without a behavioral in-group memory advantage, especially visible at PM in Figure 3. Furthermore, the recollection-related Dm for out-group faces is smaller in subjects showing a behavioral in-group than in subjects that do not show the behavioral in-group memory advantage, especially visible at CM in Figure 3.

Figure 3 also suggests that mean amplitudes for subsequently “recollected” faces gave rise to the effect observed in the recollection-related Dm reported above. Just looking at the mean amplitudes for subsequently “recollected” faces, we found that for subjects with a behavioral in-group memory advantage, mean amplitudes for subsequently “recollected” in-group faces were more positive than for subjects without the behavioral in-group memory advantage (see CM and PM for in-group faces in Figure 3). This is confirmed in MANCOVAs of mean amplitudes that yielded significant interactions for subsequently “recollected” faces but not for subsequently “familiar” faces, *p*s >.19. For subsequently “recollected” faces, an interaction of the behavioral in-group memory advantage with the run x stimulus group interaction, *F*(1,42) = 4.11, *p* = .049, was found. In the first run of the study phase, the behavioral in-group memory advantage interacted differently with the mean amplitudes for subsequently “recollected” in-group and out-group faces, *F*(1,42)  = 7.18, *p* = .020. Mean amplitudes for subsequently recollected in-group faces were more positive (*r* = .15, *p* = .33) and those for out-group faces less positive (*r* = –.14, *p* = .36) the larger the behavioral in-group memory advantage. No significant Dm effects were observed within the second run of the study phase.

**3.2.3 ERP data in the test phase.** MANCOVAs for the contrast of “recollected” and “familiar” ERPs in the FN400 time segment yielded a stimulus group x memory judgment x behavioral in-group memory advantage interaction, *F*(1,42)  = 4.68, *p* = .036. Old/new effects for in-group but not out-group faces (*p* = .78) interacted with the behavioral in-group memory advantage, *F*(1,42)  = 6.57, *p* = .028. The larger the behavioral in-group memory advantage the more positive the recollection-related old/new effect to in-group faces in the time segment of the FN400 (*r* = .37, *p* = .028; correlation for out-group faces was *r* = –.13, *p* = .78). Figure 4 illustrates this finding. For in-group faces, subjects with a behavioral in-group memory advantage showed a larger recollection-related old/new effect than subjects without a behavioral in-group memory advantage (left panel in Figure 4). No significant interactions with the behavioral in-group memory advantage were found for the contrast of ERPs to “familiar” and correctly rejected faces.

The recollection-related old/new effect in the FN400 time segment could be an early onset of the parietal old/new effect, also calculated between “recollected” and “familiar” ERPs and shown to indicate recollection processes. However additional analyses seem inconsistent with this possibility. Figure 5 shows the topographical maps of the recollection-related old/new effects for in-group and out-group faces in both time segments (300–500 ms and 600–900 ms) for subjects with and without behavioral memory advantage. This figure suggests clear differences in the spatial distribution of the recollection-related old/new effects in both time segments. We statistically tested topographical differences by separately scaling the ERPs for the old/new effects (i.e., difference waves between “recollected” and “familiar” faces) in both time segments (FN400, parietal old/new effect). For each participant, ERPs were scaled to the same overall amplitude within each condition (e.g., FN400 for in-group faces), with the average distance of the mean, derived from the individual mean ERPs for that condition, as divisor (Haig, Gordon, & Hook, 1997). MANCOVAs included the same factors as the ones reported for the test phase with the additional factor time segment (FN400, parietal old/new effect). Distinct topographies of the old/new effects in the time segments of the FN400 and the parietal old/new effect were found as indicated by significant time segment x frontal-parietal interactions for in-group, *F*(3,126)  = 3.00, *p* = .050, and out-group faces, *F*(3,126)  = 6.48, *p* = .002. This finding showed that at least some of the underlying neural sources are different for the 300–500 ms vs. 600–900 ms recollection-related old/new effects.

MANCOVAs for the parietal old/new effect yielded no significant interactions with the behavioral in-group memory advantage. For the contrast of ERPs to “recollected” and “familiar” faces, these analyses confirmed the frontal-parietal x stimulus group x memory judgment interaction, *F*(3,126)  = 3.93, *p* = .033, also found for the general analyses across all subjects reported above (see 3.1.2 and Fig. 2). Post-tests for each level of the factor frontal-parietal, including the behavioral in-group memory advantage as covariate, did also not yield any significant interaction. Instead they confirmed that irrespective of the behavioral in-group memory advantage, old/new effects for out-group faces were more positive over frontal-polar regions, *F*(1,42)  = 6.29, *p* = .036, whereas old/new effects for in-group faces tended to be more positive over parietal regions, *F*(1,44)  = 3.44, *p* = .071 (Fig. 2 and 5). Figure 5 very clearly illustrates the findings for the recollection-related old/new effects in the time segments of the FN400 and parietal old/new effect, reported in this section. Subjects with and without behavioral in-group memory advantage differ in the recollection-related old/new effect for in-group faces in the FN400 time window, but show no differences for any other comparison between in-group and out-group faces. Across all subjects, the parietal old/new effects are clearly distinct between in-group and out-group faces.

**3.2.4 Discussion of analyses with continuous measure of behavioral in-group memory advantage.** The MANCOVAs with the behavioral in-group memory advantage as independent covariate showed that the group membership of the faces affected both familiarity and recollection. Specifically, group membership affected studied faces by influencing hit rates but had no effect on memory judgments of new faces (i.e., false alarms). Faces of in-group members were more often accurately associated with feelings of familiarity and were also retrieved with more detail from the study episode. A recent study on the in-group memory advantage suggested that subjects pay more attention to in-group than out-group members during memory encoding [5]. Van Bavel and Cunningham [5], however, did not restrict the encoding time of the faces. They found that in-group faces were studied longer and were also recognized better. The in-group memory advantage in their study was thus clearly mediated by the differences in encoding time. The present study kept the encoding conditions between in-group and out-group faces constant and still found an in-group memory advantage that is not confounded with differences in encoding time. Not only were stimulus durations equated during study, but also reaction times for the attractiveness rating study task did not differ between in-group and out-group faces. The associated ERPs will therefore provide information on the neural processes underlying the in-group memory advantage independent from differences of the encoding situation.

During the initial memory encoding, neural correlates of subsequent recollection showed a clear interaction pattern with the in-group memory advantage. The larger the behavioral in-group memory advantage the larger the recollection Dm for in-group faces and the more positive mean amplitudes for subsequently “recollected” in-group faces. At the same time, the neural correlates of recollection processes during memory encoding for out-group faces were more reduced the larger the behavioral in-group memory advantage. This pattern of results suggests that memory encoding is facilitated for in-group faces, and more memory encoding resources are dedicated to in-group than out-group faces. Previous research on subsequent memory effects has shown that larger subsequent memory effects are associated with increased attention [32]–[34]. Thus, the present results provide neural evidence for the assumption that subjects pay more attention to in-group than out-group faces because they dedicate more neural resources to the encoding of in-group faces [3]–[5]. The memory encoding advantage is specific to subsequent recollection and shows that processes associated with the binding of study context to the study item were facilitated for in-group faces in subjects with an in-group memory advantage [17]. The advantage for in-group faces during memory encoding was only observed in the first run of the study phase. Only in the initial but not in the repeated encounter did subjects dedicate more encoding resources to in-group faces. This suggests that group membership does not have a constant influence on memory encoding.

Neural correlates of the behavioral in-group memory advantage were also found during memory retrieval. In the time segment of FN400, the old/new effect between “recollected” and “familiar” in-group faces was larger the larger the behavioral in-group memory effect was. This effect was not an early onset of the parietal old/new effect as confirmed with ANOVAs that compared the spatial distribution of both old/new effects (see Fig. 5). This finding suggests that subjects with a large in-group memory advantage activate frontal brain areas early during the retrieval of in-group faces to aid the recollection of these faces and associated details. This finding is in line with the observation of an in-group processing advantage in the orbitofrontal cortex [11]. The FN400 is usually associated with familiarity processes because it does normally not distinguish between “recollected” and “familiar” stimuli. In face recognition research, however, an early distinction between “recollected” and “familiar” faces is not unprecedented [35], [36].

Parietal old/new effects did not show an interaction with the in-group memory advantage. Irrespective of whether subjects recognized in-group faces more accurately than out-group faces, the parietal old/new effect for in-group faces showed a more typical parietal distribution and was larger over parietal areas whereas the parietal old/new effect for out-group faces was more positive over fronto-polar regions. This finding shows that additional brain regions are involved when retrieving out-group faces and might suggest that in-group faces are retrieved more easily, without requiring additional resources [30].

## General Discussion

Replicating previous studies, we found a behavioral in-group memory advantage following a minimal group procedure [3]–[8]. The minimal group effect in the present study was much smaller than previously reported. One major difference between previous research and the present study is the number of members of the in-group and out-group. Throughout the experiment, subjects saw 320 members each for the in-group and out-group. It is possible that the large number of group members made it harder for our subjects to identify with their specific in-group. The absence of correlations between the in-group memory advantage and measure of group affiliation provides further support for this suggestion.

Our study provides further evidence for the influence of personal factors on the minimal group effect [5]. The great individual variation in the observed in-group memory advantage suggests that not all subjects showed preferential processing of in-group faces. The felt affiliation with the personality type, at least how it was measured in our study, however, does not shed light onto these individual differences. From our study, it remains open, which factors of the individual account for the observed in-group advantage.

The present study provides first evidence about the neural processes of memory encoding and retrieval that underlie the in-group memory advantage. Considering the two sub-processes of recognition memory, only neural processes of recollection but not familiarity were influenced by group membership. The absence of familiarity effects in the ERPs could be due to a power problem in the ERPs because group membership did affect behavioral measures of familiarity.

The findings on neural processes of the minimal group effect can be divided into two groups: true correlates of the behavioral in-group memory advantage and effects that are independent of the behavioral in-group memory advantage. The first can be explained more easily than the latter. Correlations of the behavioral in-group memory advantage were found during memory encoding where subsequently recollected in-group faces elicited more positive amplitudes and led to a larger Dm effect. This finding confirms the assumption that during memory encoding in-group faces receive more attention and cognitive resources whereas out-group faces do not and are thus disregarded to some extend [3]–[5]. We also found correlates of the in-group memory advantage during memory retrieval. Especially during the early retrieval stages, in-group faces were favored and elicited larger memory recollection-related retrieval effects over frontal areas. Frontal brain areas were suggested to be involved in segregating more relevant and salient in-group from less relevant out-group faces [11]. This early detection of group membership during memory retrieval might facilitate the retrieval of the face from memory.

Independent of the behavioral in-group memory advantage, all subjects—even those that did not favor in-group faces during memory encoding and did not show better memory performance for in-group faces—activated distinct brain areas when recollecting in-group and out-group faces as indicated by the difference distributions of the parietal old/new effect. In our experiment, group membership was indicated by the background during encoding and also retrieval. It is therefore possible that group membership influenced memory retrieval independent from memory encoding because during retrieval subjects did not have to rely on their memory about group membership. Instead, group membership became a visible characteristic of the faces and could be thought to have triggered a specific retrieval mechanism. Whereas all in-group faces were retrieved using the typically involved brain areas, out-group faces activated frontal brain areas in addition. The involvement of frontal brain areas might indicate that subjects used more cognitive control like post-retrieval monitoring during the retrieval of out-group faces [37], [38].

The in-group memory advantage observed for the parietal old/new effect across all subjects is the only finding that shows parallels to our previous study on the other-race effect [30]. This similar finding suggests that memory retrieval can be influenced in the same way by the race of a face and social classification based on personality type. Given the interpretation above, that in the present experiment group membership became a visible characteristic during memory retrieval, the parallels between the in-group memory advantage and the other-race effect are not surprising. In both cases, subjects can be thought to use the group-identifying feature (i.e., race or background color) to bias their memory retrieval. In this context, the differences between the memory effects for face classifications based on visible features (own-race vs. other-race) and non-visible features (own vs. other personality type) seem more surprising. Especially during memory encoding the findings of the present study and the previous other-race effect study diverge. Previously we found less positive amplitudes for subsequently remembered own-race as compared to other-race faces [30], whereas here subsequently remembered in-group faces elicited more positive amplitudes than out-group faces. Interestingly, our previous result for own-race faces resembled findings in a study on car expertise [39] pointing to an influence of increased perceptual experience on the processing of own-race as compared to other-race faces. Acknowledging the limits of a cross-experiment comparison, the findings of the present and the two previous studies suggest that memory for faces that can be classification based on visible characteristics like race or age can be influenced by the extensive perceptual experience people have for these faces. In contrast, recognizing faces that are classified based on invisible characteristics like personality type cannot profit from perceptual experience and relies more on top-down processes activated by the knowledge we have gained about these faces [10].

## Conclusion

The present study showed that group membership shapes face recognition processes and that some individuals are more susceptible to the influence of group membership than others. We provided first neural evidence for the assumption that in-group faces receive more attention during memory encoding and showed that group membership biased early memory retrieval processes. Knowing that a face belongs to the own or the other group triggered different brain areas during memory retrieval and led to increased memory performance for in-group faces. However, it did not correlate with the size of the in-group memory advantage. The comparison of the in-group memory advantage obtained with the minimal group procedure to the other-race effect, another phenomenon that relies on social classification of faces, suggests that top-down processes play a larger role for the in-group memory advantage and that perceptual experience shapes the other-race effect to a greater extent.

## Supporting Information

File S1 **All 44 items used in the bogus personality test.**
(DOCX)

File S2 **Questionnaire about the affiliation participants felt toward their personality type.**
(DOCX)
