# Supplementary material for: Neural Correlates of the In-Group Memory Advantage on the Encoding and Recognition of Faces
Source: PLoS One. 2013 Dec 17;8(12):e82797. doi: 10.1371/journal.pone.0082797 (PMC3866141; doi:10.1371/journal.pone.0082797)
Supplement: File S1 — All 44 items used in the bogus personality test. (DOCX) [file pone.0082797.s001.docx]

**Supporting Information S1**

1. I am someone who is talkative.
2. I am someone who tends to find fault with others.
3. I am someone who does a thorough job.
4. I am someone who is depressed, blue.
5. I am someone who is original, comes up with new ideas.
6. I am someone who is reserved.
7. I am someone who is helpful and unselfish with others.
8. I am someone who can be somewhat careless.
9. I am someone who is relaxed, handles stress well.
10. I am someone who is curious about many different things.
11. I am someone who is full of energy.
12. I am someone who starts quarrels with others.
13. I am someone who is a reliable worker.
14. I am someone who can be tense.
15. I am someone who is ingenious, a deep thinker.
16. I am someone who generates a lot of enthusiasm.
17. I am someone who has a forgiving nature.
18. I am someone who tends to be disorganized.
19. I am someone who worries a lot.
20. I am someone who has an active imagination.
21. I am someone who tends to be quiet.
22. I am someone who is generally trusting.
23. I am someone who tends to be lazy.
24. I am someone who is emotionally stable, not easily upset.
25. I am someone who is inventive.
26. I am someone who has an assertive personality.
27. I am someone who can be cold and aloof.
28. I am someone who perseveres until the task is finished.
29. I am someone who can be moody.
30. I am someone who values artistic, aesthetic experiences.
31. I am someone who is sometimes shy, inhibited.
32. I am someone who is considerate and kind to almost everyone.
33. I am someone who does things efficiently.
34. I am someone who remains calm in tense situations.
35. I am someone who prefers work that is routine.
36. I am someone who is outgoing, sociable.
37. I am someone who is sometimes rude to others.
38. I am someone who makes plans and follows through with them.
39. I am someone who gets nervous easily.
40. I am someone who likes to reflect, play with ideas.
41. I am someone who has few artistic interests.
42. I am someone who likes to cooperate with others.
43. I am someone who is easily distracted.
44. I am someone who is sophisticated in art, music, or literature.
