# Supplementary material for: Neural Correlates of the In-Group Memory Advantage on the Encoding and Recognition of Faces
Source: PLoS One. 2013 Dec 17;8(12):e82797. doi: 10.1371/journal.pone.0082797 (PMC3866141; doi:10.1371/journal.pone.0082797)
Supplement: File S2 — Questionnaire about the affiliation participants felt toward their personality type. (DOCX) [file pone.0082797.s002.docx]

**Supporting Information S2**

Please indicate your agreement/disagreement with each of the following statements, using the scale below. Please write your rating in the blank to the left of each statement.

1----------2----------3----------4----------5----------6----------7

Strongly Strongly

Disagree Agree

_____ 1. During the experiment, I thought often about my personality group.

_____ 2. During the experiment, I felt a strong attachment to other people in my

personality group.

_____ 3. My personality group is important to me.

_____ 4. I believe that my future is tied to my personality group.

_____ 5. My personality group is an important part of who I am.

_____ 6. Overall, my personality group has very little to do with how I feel about myself.

_____ 7. I have a strong sense of belonging to my personality group.
